# Mechanical-Enhanced Porous Silk-Based Cryogenic Microneedles for Cell Thawing/Revival in the Gastric Wall

**DOI:** 10.3390/polym18131654

**Published:** 2026-07-03

**Authors:** Zhiwei Yin, Limin Zhang, Rui Shi, Xin Xia, Zhaoxin Wang, Ling Li, Zhuo Chen

**Affiliations:** 1 The University of Hong Kong Shenzhen Institute of Research and Innovation, Shenzhen 518048, China; robbies@hku.hk (Z.Y.); zlimin61@hku.hk (L.Z.); 2 Molecular Science and Biomedicine Laboratory (MBL), State Key Laboratory of Chemo and Biosensing, College of Chemistry and Chemical Engineering, College of Environmental Science and Engineering, Aptamer Engineering Center of Hunan Province, Hunan University, Changsha 410082, China; 3 School of Materials and Environmental Engineering, Changsha University, Changsha 410022, China; 4 Zhejiang Cancer Hospital, Hangzhou Institute of Medicine (HIM), Chinese Academy of Sciences, Hangzhou 310022, China

**Keywords:** cell therapy, porous silk scaffold, cryogenic microneedle, β-sheet transformation, gastric wall penetration

## Abstract

Cell therapies for gastric disorders lack minimally invasive delivery platforms that preserve cell viability during storage and enable effective tissue penetration, owing to the high toughness and harsh environment of the gastric wall. Herein, we developed a mechanically reinforced, porous silk-based cryogenic microneedle (silk-cryoMN) platform for in situ cell delivery to the gastric wall. The optimized 1.5% (*w*/*v*) silk scaffolds exhibited interconnected pores (24.4 ± 7.9 μm, ~81% porosity), a compressive strength (422.8 ± 73.4 MPa), and a 3.4-fold increase in β-sheet content. The silk-cryoMNs showed greater thermal stability than H_2_O-cryoMNs, maintaining structural integrity for over 60 s at room temperature. With a cryopreservation medium containing 100 mM sucrose and 2% DMSO, post-thaw cell viability exceeded 80% after 11 days of freezing, and most cells were released within 1 h. Furthermore, ex vivo studies confirmed penetration of porcine gastric tissue to depths of 422–448 μm within 30 s. These results suggest that the platform may address several translational barriers, including tissue penetration, handling stability, and cell viability preservation. Further in vivo studies and long-term safety evaluations are needed before clinical translation can be considered.

## 1. Introduction

Cell therapy has emerged as a transformative modality for treating diverse diseases, including cancer and tissue injuries [1,2,3]. Unlike traditional small-molecule drugs that primarily manage symptoms, cell-based therapeutics offer the potential to restore normal tissue function, modulate immune responses, and promote long-term regeneration [4,5,6]. The therapeutic cells can home to sites of injury and secrete paracrine factors that suppress inflammation, promote angiogenesis, and stimulate endogenous repair mechanisms [7,8,9]. Such cells have shown remarkable promise in both preclinical studies and clinical trials [10].

Despite these advances, the successful clinical translation of cell therapies hinges on the development of effective delivery systems. The route of administration profoundly influences cell survival, engraftment, biodistribution, and therapeutic efficacy. Systemic delivery via intravenous injection exposes cells to mechanical shear stress, immune surveillance, and rapid clearance by the lungs, liver, and spleen [11,12]. Therefore, local delivery directly to the site of injury or disease offers several advantages: reduced cell loss, lower risk of ectopic distribution, decreased off-target effects, and the ability to achieve therapeutic effects with fewer cells [13]. Particularly, local delivery to the gastric wall could enable targeted cell therapy while minimizing systemic exposure and adverse effects [14,15]. However, delivering functional cells to the gastric wall presents unique and significant challenges [16]. Conventional delivery methods for gastrointestinal cell therapy include endoscopic needle injection and surgical transplantation [17]. The procedural complexity, biohazard generation, and high healthcare costs remain major obstacles to clinical adoption [18,19].

Microneedle (MN) technology has evolved into a versatile platform for minimally invasive delivery of biologics and living cells across diverse tissue barriers [20]. Comprising arrays of micron-scale projections, MNs penetrate superficial tissue layers without engaging nociceptors, thereby enabling painless, efficient, and patient-friendly delivery [21,22,23]. While most advanced applications have focused on transdermal delivery of vaccines, proteins, nucleic acids, and even viable cells, recent studies have extended this paradigm to cryogenic microneedles (cryoMNs), which encapsulate cells within ice-based matrices for direct tissue implantation [24,25]. Although pure water-based cryoMNs offer simplicity, their rapid melting (within 30~60 s) and poor mechanical integrity render them unsuitable for penetrating the mechanically resilient gastric wall [26]. Thus, extending thermal stability and augmenting structural rigidity are essential prerequisites for gastric application.

Silk fibroin possesses remarkable mechanical properties, biocompatibility, tunable biodegradability, and the ability to stabilize labile biologics during processing and storage [27]. During freezing, silk fibroin undergoes a conformational transition from random coil and α-helix to β-sheet structures, creating a reinforcing protein network within the ice matrix [28]. Such silk-reinforced ice exhibits greater mechanical strength and fracture toughness than ice alone, enabling reliable penetration of the gastric wall. Upon lyophilization, the silk network forms a porous scaffold with high interconnectivity that facilitates cell loading, nutrient diffusion, waste exchange, and rapid cell release [29]. The porous architecture also promotes cell survival by providing physical support and enabling uniform distribution within the microneedle [30,31]. Furthermore, the silk matrix can be functionalized with cell-adhesive motifs or growth factors to enhance cell engraftment and function [32].

**Figure 1:**
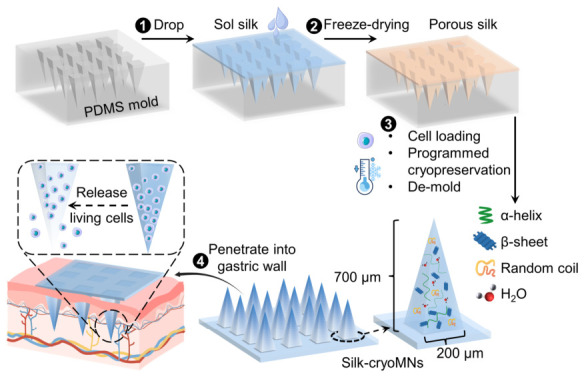
Schematic illustration of the silk-cryoMN fabrication process and in situ cell thawing/revival. 1, Silk solution is cast into a PDMS mold; 2, after degassing, frozen at −20 °C overnight and lyophilized; 3, the cells and cryopreservation medium mixtures were transferred into the porous silk, after programmed cryopreservation, demolded to obtain silk-cryoMNs; 4, the silk-cryoMNs penetrate the gastric wall for cell thawing/revival.

In this study, we present the rational design and proof-of-concept evaluation of a mechanically enhanced porous silk-based cryogenic microneedle (silk-cryoMN) platform for in situ delivery of functional cells to the gastric wall (Figure 1). We systematically optimized the cryopreservation formulation to maximize post-thaw viability across multiple cell types, characterized the porous microstructure, and demonstrated that ~81% porosity with high connectivity enables efficient cell release. Such silk-cryoMNs showed enhanced mechanical strength and were assessed for their ability to penetrate ex vivo porcine gastric tissue. Moreover, the loaded cells showed good viability throughout silk-cryoMN fabrication, lyophilized storage, and delivery. Collectively, this work represents the new cryoMN system specifically engineered for gastric wall delivery, distinguished by its β-sheet-reinforced mechanical strength, interconnected porous architecture, and optimized cryoprotection. Thus, the silk-cryoMN platform is a biocompatible, scalable, and functionally effective platform for gastric-targeted cell therapies with significant implications for gastric cancer immunotherapy, peptic ulcer healing, and gastric tissue engineering.

## 2. Materials and Methods

### 2.1. Synthesis and Fabrication of Silk-cryoMNs

The sol-silk solution [33] was prepared by degumming silkworm cocoons three times in 0.05 M NaHCO_3_ at boiling temperature, followed by dissolution in 9.3 M LiBr at 60 °C for 1 h, dialysis (3 kDa cutoff) against deionized water for 24 h, and centrifugation at 4000 rpm for 30 min to remove insoluble residues. The sol-silk solution was then diluted to desired concentrations, dropped into a PDMS microneedle mold, degassed, frozen at −20 °C overnight, and lyophilized to obtain porous silk scaffolds. Next, the cells and cryopreservation medium mixtures were transferred into the porous silk after programmed cryopreservation (4 °C for 1 h, −20 °C for 2 h, −80 °C overnight, liquid nitrogen for long-term storage) [34]. Once ready, demold for isolation of silk-cryoMNs, which need to penetrate into the stomach wall rapidly.

### 2.2. Characterization of Silk-cryoMNs

All silk scaffolds or cryoMNs were characterized using similar methods of characterization. Specifically, the morphology of scaffolds was observed by scanning electron microscopy (SEM, TESCAN, Czech) at an accelerating voltage of 20 kV and 30 s gold spraying. For cell-loaded scaffolds, samples were treated with 90 s gold spraying, followed by SEM-EDS mapping for visualization of element distribution. Mechanical properties of silk scaffolds (1 cm × 1 cm × 1 cm) were determined using a testing machine (HYC-2011, Hongjin, China) equipped with a 5000 N load cell. Specimens were compressed at a speed of 20 mm/min. Compressive strength (MPa) was computed accordingly from the generated stress–strain curves [35].

To investigate the thermal stability of porous silk scaffolds, the 1.5% silk scaffolds were immersed in PBS supplemented with 10% FBS at 37 °C for 1, 2, 3, 4, and 5 weeks. Weight loss was calculated as

$$Equation:Weight\;loss\left(\%\right)=\frac{∆m}{m_{0}}\times 100\%$$

where ∆m is the amount of change in weight, and m0 is the initial value of weight.

Circular dichroism (CD) spectra were measured at room temperature by using a Circular Dichroism Spectrometer MOS-500 (Bio-Logic Science Instruments, France). The sol-silk or porous silk was prepared at A280 = 0.1. The temperature was maintained at 25 °C throughout. A 1 mm quartz cuvette was used for all CD spectra. Data were recorded from 250 nm to 190 nm with a 1 nm sampling interval. The final spectra were the average of three repeated experiments, and the background was subtracted. The CD spectra were analyzed using the online tool BESTSEL (https://bestsel.elte.hu/index.php, accessed on 21 April 2026) to obtain statistics on protein secondary structure content [36].

Infrared spectroscopy (Plastic Analyzer, SHIMADZU, Japan) provided spectra containing signals from the functional groups in the sol-silk or porous silk.

### 2.3. Micro-CT and Modeling of Silk-cryoMNs

X-ray CT scans were performed with the Xradia 515 Versa (Zeiss, Germany), and CT data were analyzed and modeled using Dragonfly 2022.2 [37]. A cylindrical volume was scanned in silk-cryoMNs. We chose a smaller sub-volume of 10 mm^2^ × 1 mm for the calculation of porosity, diameters, and connectivity among the cells. The permeability analysis was performed using path modeling in a liquid environment for the visualization of the diffusion rate. For angle modeling, pore orientation was analyzed by calculating the angle between the major axis of each pore and the X-, Y-, or Z-axis using a 3D ellipse-fitting algorithm in Dragonfly 2022.2. The angular frequency distribution was plotted to assess pore alignment isotropy.

### 2.4. Cell Lines and Cell Cultures

GES-1 (human gastric mucosal epithelial cells, ATCC, Manassas, USA) was cultured in RPMI-1640 (Gibco, USA) supplemented with 10% FBS (Gibco, USA) and 1% P/S (Gibco, USA). NIH-3T3 (mouse embryonic fibroblasts, Pricella, Wuhan Procell Biotechnology Co., Ltd., China) and HeLa (human cervical cancer cells, Pricella, Wuhan Procell Biotechnology Co., Ltd., China) were cultured in high-glucose DMEM (Gibco, USA) supplemented with 10% FBS and 1% P/S. All cells were maintained at 37 °C with 5% CO_2_. The HeLa-eGFP cells were fabricated by infecting with eGFP-lentivirus, which was imaged under a laser scanning confocal microscope (Nikon, Japan) or a fluorescence imaging system (IVIS Lumina Xr, USA).

### 2.5. Structure Thermal-Stability Analysis

H2O-cryoMNs and silk-cryoMNs were captured at 10 s, 20 s, 30 s, 40 s, 50 s, and 60 s for the visualization of melting. Meanwhile, the real-time temperature was recorded by an IR thermal imaging camera (FOTRIC 365, China). Briefly, the cryoMNs were placed and recorded for 5 min at room temperature, and the temperature change and time at 0 °C were calculated.

### 2.6. Live/Dead Staining for Cell Viability and Release

To optimize the cryopreservation medium. The solutions with gradient concentrations of sucrose were prepared: 50 mM, 100 mM, 150 mM, 200 mM, and 250 mM. At the same time, the gradient concentrations of DMSO were prepared with volume percentages of 1%, 1.5%, 2%, 2.5%, and 5%, respectively. HeLa cells were frozen with sucrose or DMSO solutions of the above concentrations under programmed cryopreservation. The cells were resuscitated after 3 days of freezing. The cells were then stained for live/dead using a Calcein/PI cell viability assay kit and captured using a confocal high-content spinning disk (Perkin Elmer, Opera Phenix Plus, USA) to determine the optimal concentration of sucrose or DMSO for use [38].

The GES-1 and NIH-3T3 cells were frozen with 100 mM sucrose and 2% DMSO solutions under programmed cryopreservation. The cells were resuscitated after 11 days of freezing. The released cells from silk-cryoMNs were stained for live/dead using a Calcein/PI cell viability assay kit (Beyotime, China) to confirm the revived cell state and counted for calculation of the cell release rate from silk-cryoMNs.

### 2.7. Ex Vivo Studies

The proof-of-concept evaluation of silk-cryoMNs in situ using porcine gastric tissue. Briefly, the silk-cryoMNs were retrieved from −80 °C, transferred onto dry ice to prevent premature thawing, and gently demolded. Following incubation at room temperature for varying durations (10 s, 30 s, 50 s, and 70 s), the microneedles were pressed onto the surface of the gastric wall and maintained under gentle pressure for 10 min to allow for tissue penetration. The stabbed gastric tissue was fixed in a 4% paraformaldehyde solution, followed by gradient dehydration with alcohol of different concentrations, clearing, and embedding in paraffin blocks. Samples were sliced and stained with hematoxylin and eosin (H&E) according to the general protocol. The penetration depth was measured from the H&E images using ImageJ 1.43u software.

### 2.8. Statistical Analysis

All quantitative data are presented as mean ± standard deviation (n ≥ 3 data points per experiment). All data were analyzed using one-way analysis of variance (ANOVA) with Tukey’s test to determine the differences between groups using GraphPad Prism 7. A value of *p* > 0.05 was considered not significant (n.s.), while a value of *p* < 0.05 was considered statistically significant. The significance level is presented as * *p* < 0.05, ** *p* < 0.01.

## 3. Results

### 3.1. Fabrication and Characterization of β-Sheet-Rich Isotropic-Porous Silk-cryoMNs

**Figure 2:**
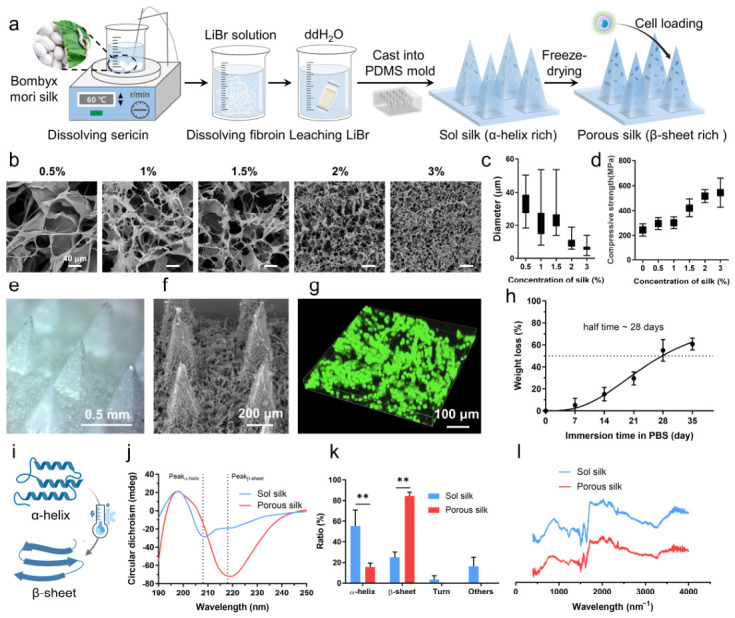
Characterization of the porous silk scaffold as a cryoMN framework with tunable pore size, compressive strength, and high β-sheet content for cell loading and mechanical reinforcement. (**a**). Schematic illustration for the synthesis of sol-silk and porous silk. (**b**–**d**). SEM ((**b**), scale bar = 40 μm) image, the pore diameters ((**c**), n = 30), and compressive strength ((**d**), n = 3) of porous silk scaffold at different concentrations. (**e**–**g**). Gross ((**e**), scale bar = 0.5 mm) and SEM ((**f**), scale bar = 200 μm) images of the 1.5% silk microneedle scaffold, and confocal image ((**g**), scale bar = 100 μm) of the HeLa-eGFP-loaded scaffold. (**h**). Weight loss of 1.5% silk microneedle scaffold after immersion in PBS at 37 °C over time, n = 5. (**i**). Schematic transformation from α-helix to β-sheet after freeze. (**j**–**l**). Circular dichroism spectra (**j**), content statistics of protein secondary structure (**k**), and FTIR spectra (**l**) of sol-silk and porous silk. The data shown from representative sample and presented as means ± s.d.; the asterisk indicates a statistically significant difference, ** *p* < 0.01.

In situ cell thawing/revival was expected to drive research into the next generation of cellular administration modes. The silk was selected as the framework for cryoMNs due to its biocompatibility, biodegradability, low cost, low immunogenicity, and good mechanical properties [39,40,41]. Firstly, natural cocoons were processed by dissolving sericin, followed by dissolving fibroin to obtain sol-silk (Figure 2a). In order to optimize the ideal silk concentrations, the 0.5%, 1%, 1.5%, 2%, and 3% silk porous frameworks were fabricated. The morphology and mechanical properties of silk porous frameworks were characterized using scanning electron microscopy (SEM, Figure 2b) and compression force applied to the flat surface of the frameworks, followed by strength calculation (Figure 2c,d and Figure S1). The data showed that the pore diameters decreased and compressive strength increased with rising silk concentrations. Due to the requirement for favorable cell size adaptation and adequate mechanical strength, the 1.5% silk concentration was selected as optimal, offering a balance between pore diameter (24.4 ± 7.9 μm) and compressive strength (422.8 ± 73.4 MPa). Next, the 1.5% silk microneedle scaffold was fabricated using a PDMS mold. The morphology and microstructure of silk microneedle scaffolds were characterized using optical microscopy (Figure 2e), SEM (Figure 2f), and 3D confocal imaging (Figure 2g). The data illustrated that the surface of quadrangular pyramid microneedles (height of 700 μm and base width of 200 μm) was mainly composed of the continuous pore structure, and the HeLa-eGFP cells (Figure S2) could evenly distribute within the pores, and the maximum cell loading reached 3 × 10^6^ cells per scaffold (Ø 1 cm × 5 mm, containing a 10 × 10 array of pyramidal microneedles on its surface, 5 × 10^6^ cells in 0.5 mL medium for loading) via macroscopic fluorescence quantification (Figure S3).

Given that β-sheet transformation can enhance the chemical/physical stability of proteins [42,43]. The weight loss of the porous silk scaffold was measured, and the calculated half-time of 28 days meets the requirement for application, as expected (Figure 2h). During freezing, silk fibroin undergoes a conformational transition from random coil and α-helix to β-sheet (Figure 2i). Such transformation of protein secondary structures was proved by circular dichroism spectra and their analysis (Figure 2j,k). The data showed that the enhanced peak_β-sheet_ and the reduced peak_α-helix_ were found in the porous silk scaffold, which exhibited a 3.4-fold higher ratio of β-sheet and a 3.5-fold lower ratio of α-helix than sol-silk. Moreover, the turn and random coil are not represented in the porous silk scaffold. Fourier transform infrared (FTIR) spectroscopy showed no significant chemical changes (Figure 2l). Therefore, the porous silk scaffold represents a suitable cryoMN framework, offering tunable pore size and compressive strength, as well as a high β-sheet proportion that facilitates both cell loading and mechanical reinforcement.

**Figure 3:**
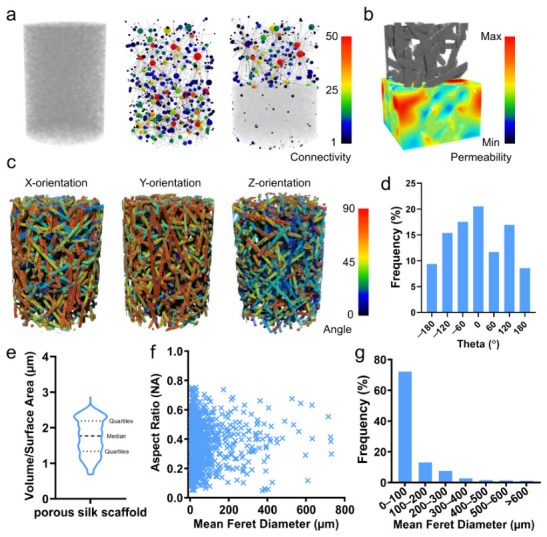
Pore structure analysis of the silk microneedle scaffold. a-c. Micro-CT images and modelling of the porous 1.5% silk microneedle scaffold showing porous structure and well-connectivity (**a**), good permeability (**b**), and high isotropy at XYZ-orientation (**c**). (**d**–**g**). Orientation angle (**d**), volume/surface area (**e**), aspect ratio (**f**), and frequency of pores in different diameters (**g**) of pores in the scaffolds, showing that most pores exhibited an oblong shape, acceptable diameters, and isotropic structure. The data shown from representative sample and presented as means ± s.d.

The internal porous structures of the 1.5% silk microneedles were further investigated using X-ray micro-computed tomography (Micro-CT), which provides critical guidance for cell colonization and release. The micro-CT analysis revealed the detailed 3D porous network within the silk scaffold. The scaffold exhibited interconnected pores (24.4 ± 7.9 μm, ~81% porosity) with a mean connectivity of ~3.96 per pore (Figure 3a) and good permeability (Figure 3b), which are essential for uniform cell distribution and efficient exchange. The simulated fibers exhibited orientation-independent pore architecture along the X, Y, and Z directions (Figure 3c,d), indicating an isotropic structure. Moreover, most pores exhibited a volume/surface area of 1.74 ± 0.49 µm (Figure 3e), an oblong shape with an aspect ratio of ~0.5 (Figure 3f), and a frequency of ~72.2% within the 0–100 µm range (Figure 3g). These structural parameters collectively support favorable conditions for cell infiltration, uniform loading, and subsequent release.

### 3.2. Thermal-Stability Analysis and Cryopreservation Medium Optimization of Silk-cryoMNs

**Figure 4:**
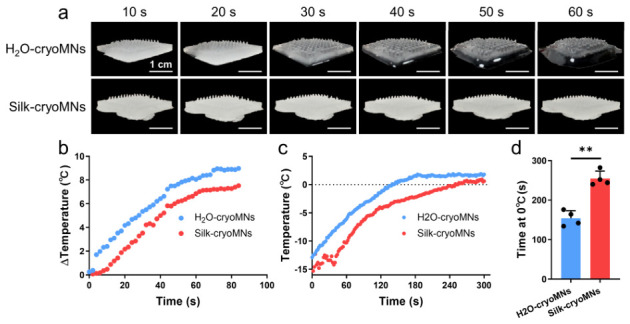
Structural stability analysis of H_2_O-cryoMNs and silk-cryoMNs. (**a**), H_2_O-cryoMNs and silk-cryoMNs over time, scale bar = 1 cm. (**b**,**c**). The temperature change (**b**) and real-time temperature (**c**) curves over time, and the statistics of time at 0 °C ((**d**), n = 4) of H_2_O-cryoMNs and silk-cryoMNs. The data shown from representative sample and presented as means ± s.d.; the asterisk indicates a statistically significant difference, ** *p* < 0.01.

Cell delivery fails once the cryoMNs melt or cells lose viability; thus, ensuring acceptable thermal stability and medium optimization was essential for maintaining structural integrity and cell viability during storage and handling [44]. Consequently, thermal-stability analysis and cryopreservation medium optimization of the silk-cryoMNs were performed. In general, 1 min of stability upon take-out from the cryogenic environment is sufficient for the cryoMNs to remain intact before use. The H_2_O-cryoMNs exhibited progressive surface melting and structural collapse at 30 s, whereas silk-cryoMNs maintained their intact geometry throughout the observation period, indicating superior structural stability (Figure 4a). Next, the real-time temperature of cryoMNs was detected (Figure 4b,c). The data demonstrated that the silk-cryoMNs exhibited a slower temperature rise upon removal from the cryogenic environment compared to H_2_O-cryoMNs, maintaining sub-zero temperatures for a longer duration (Figure 4d), which further supports silk-cryoMNs’ enhanced thermal stability and suitability for handling before administration.

**Figure 5:**
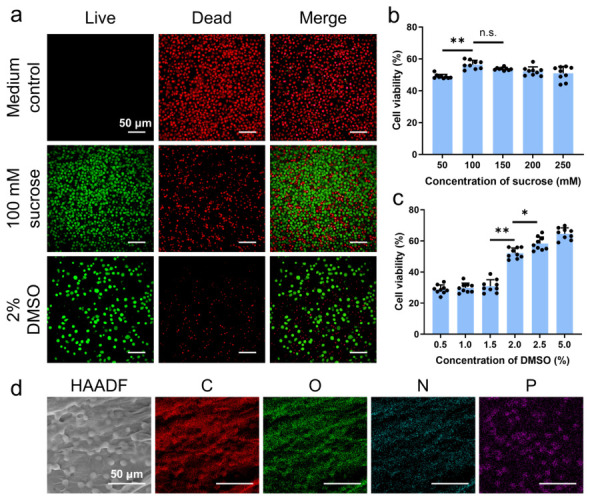
Optimization of cryopreservation medium. (**a**). The confocal image of revived HeLa with medium, 100 mM sucrose, and 2% DMSO, scale bar = 50 μm. (**b**,**c**), The revived HeLa viability with different concentrations of sucrose ((**b**), n = 9) and DMSO ((**c**), n = 9). (**d**). SEM-EDS mapping of silk-cryoMN-loaded living cell and cryopreservation medium mixture, showing HAADF (high-angle annular dark-field) image and elemental maps of carbon (C), oxygen (O), nitrogen (N), and phosphorus (P), scale bar = 50 µm. The data shown from representative sample and presented as means ± s.d.; the asterisk indicates a statistically significant difference, * *p* < 0.05, ** *p* < 0.01, n.s. not significant.

Given that the primary reason for cell death after cryopreservation is membrane damage caused by ice crystallization [45,46]. The vitrification approach, while effective in preventing ice formation, introduces the risk of cryoprotectant-induced toxicity and osmotic stress. Therefore, strategies that minimize ice crystallization without relying on high cryoprotectant concentrations are highly desirable. Sucrose and DMSO were selected as cryoprotectants due to their ability to inhibit ice crystal formation and stabilize cell membrane integrity. First, the revived HeLa viability with various concentrations of sucrose or DMSO was assessed using live/dead staining (Figure 5a, Figures S4 and S5). The statistics indicated that moderate concentrations of 100 mM sucrose and 2% DMSO could effectively maintain post-thaw cell viability, while avoiding excessive concentrations that caused toxic effects (Figure 5b,c). Because the potential cytotoxicity of DMSO requires a careful balance between achieving favorable post-thaw viability and using the lowest effective concentration. Moreover, the SEM-EDS mapping revealed that the intact cells with unbroken cell membrane (marked by P-element) were present on the resuscitated silk-cryoMNs (Figure 5d); such cell morphology would be considered a living cell [47]. Collectively, these data above confirm that the silk fibroin matrix effectively retards heat transfer and delays ice melting, thereby providing silk-cryoMNs sufficient thermal stability to preserve structural integrity and cell viability during handling.

### 3.3. The Proof-of-Concept Evaluation of Silk-cryoMNs In Situ

**Figure 6:**
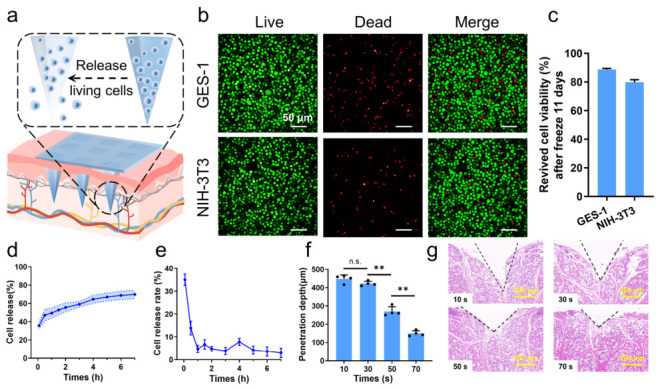
Proof-of-concept evaluation of silk-cryoMNs in situ. (**a**). Schematic of the silk-cryoMNs for cell thawing/revival in the gastric wall. (**b**,**c**). The confocal image and statistics of cell viability of the revived human gastric mucosal epithelial cells (GES-1) and the revived mouse embryonic fibroblasts (NIH-3T3) following cryopreservation for 11 days using an optimized cryopreservation medium (100 mM sucrose and 2% DMSO), scale bar = 50 μm. (**d**,**e**). The cell release ((**d**), n = 3) and cell release rate ((**e**), n = 3) curves of silk-cryoMNs. (**f**,**g**). The penetration depth analysis ((**f**), n = 4) and H & E staining ((**g**), scale bar = 200 μm) of porcine gastric tissue after piercing with silk-cryoMNs that had been kept at room temperature for 10 s, 30 s, 50 s, and 70 s. The data shown from representative sample and presented as means ± s.d.; the asterisk indicates a statistically significant difference, ** *p* < 0.01, n.s. not significant.

To confirm whether the silk-cryoMNs could effectively release revived living cells and penetrate into the gastric wall, a proof-of-concept evaluation was performed using ex vivo porcine gastric tissue (Figure 6a). First, the human gastric mucosal epithelial cells (GES-1) and the mouse embryonic fibroblasts (NIH-3T3) were selected to investigate cell delivery and verify cell universality. After 11 days of cryopreservation, the silk-cryoMNs were retrieved, thawed, and placed into a 6-well plate containing 1 mL medium. The released cells were then collected from the medium for live/dead staining (Figure 6b). The data showed that the cell viability exceeded 80% under a 100 mM sucrose and 2% DMSO mixture (Figure 6c). The intact cells were also detected by SEM (Figure S6). Subsequently, the cell release and release rate were calculated (Figure 6d,e). Most cells were released within 1 h, and the cell release rate decreased markedly after 1 h. Moreover, the stabbed gastric walls were sliced and stained with hematoxylin and eosin to observe the penetration depth in situ (Figure 6f,g). The data illustrated that penetration depth decreased with application time, reaching 422.6~448.2 μm within 30 s and remaining at 149.6 ± 14.7 μm in 70 s, confirming effective insertion of silk-cryoMNs into the gastric wall. Collectively, these results demonstrated that the silk-cryoMN platform enables effective cell delivery with high post-thaw viability, sustained release, and adequate tissue penetration, supporting its potential for cellular therapies in the gastric wall.

## 4. Discussion

The silk-cryoMN platform features a mechanically reinforced, well-interconnected porous architecture that facilitates cell thawing and revival within the gastric wall. This functionality derives from three synergistic mechanisms. First, freezing induces a conformational shift of silk fibroin from random coil/α-helix to β-sheet, forming a crosslinked protein network within the ice matrix. This network simultaneously boosts compressive strength and slows heat transfer, allowing silk-cryoMNs to remain intact for >60 s at room temperature, whereas water-based cryoMNs collapse within 30 s. Notably, this 60 s window applies to ex vivo handling at room temperature (~25 °C) after cryogenic retrieval. We acknowledge that this window may be insufficient for complex endoscopic procedures, as the actual intra-body stability at 37 °C is expected to be shorter. Nevertheless, the >100% extension relative to water-based cryoMNs demonstrates the material improvement achievable through β-sheet reinforcement. This thermal defense effect is likely attributable to physical crosslinking of β-sheet within the ice matrix. Further optimization (incorporating thermal insulation layers) may help to extend the handling window for practical clinical use. Compared to previously reported ice-based cryoMNs, our silk-reinforced system offers superior mechanical strength and thermal stability, which are essential for penetrating the tougher gastric wall. Second, the highly interconnected porous architecture facilitates both uniform cellular loading and rapid post-thaw release, achieving >80% viable cell release within 1 h. Last, the optimized DMSO (inhibition of intracellular ice formation) and sucrose (stabilization of cell membrane integrity) act in a complementary combination, yielding favorable post-thaw viability after 11 days of cryopreservation.

The gastric wall is the primary site of gastric cancer and peptic ulcers, making it a clinically relevant target for local cell therapy. Compared to conventional gastric cell delivery methods (endoscopic needle injection and surgical transplantation), the silk-cryoMN platform offers several distinct advantages. Endoscopic injection is invasive, requires specialized equipment and trained personnel, and risks uneven cell distribution, bleeding, or perforation. Surgical transplantation is even more invasive, costly, and associated with significant morbidity. In contrast, our platform is minimally invasive, painless (micron-scale projections avoid nociceptors), and can be applied in a single rapid step without complex instrumentation. Relative to previously reported ice-based cryoMNs, which suffer from rapid melting and poor mechanical integrity, the silk-reinforced cryoMNs provide extended handling time and sufficient mechanical strength to reliably penetrate the mechanically resilient gastric wall, as confirmed by ex vivo porcine tissue studies. The release kinetics (Figure 6d,e) confirmed >80% viable cell release within 1 h upon thawing. The absence of visible cell clusters in H&E images (Figure 6f,g) is likely due to the ex vivo model: lack of active perfusion for immediate cell adhesion and potential loss of non-adherent cells during histological processing. Importantly, the cells delivered into the submucosal layer (422~448 μm) are protected from digestion, as they are deposited beneath the epithelium and away from the gastric lumen. Moreover, the current ex vivo model does not provide evidence of cell migration or survival within the gastric wall; future in vivo studies using labeled cells are required to confirm successful cell delivery and tissue integration.

Despite these promising results, in vivo validation in large animal models (e.g., porcine or canine) is urgently needed to assess long-term scaffold degradation, cell engraftment, therapeutic efficacy, and immune responses under physiological conditions, including peristalsis and gastric acid secretion. Furthermore, the 60 s handling window, while improved over water-based cryoMNs, is not yet sufficient for practical clinical endoscopic procedures; further optimization is required before clinical translation can be considered. Moreover, future studies could extend to primary human gastric cells, stem cells, or therapeutic immune cells (e.g., CAR-T cells) for a wide variety of disease treatments. Additionally, the silk matrix could be further functionalized with bioactive ligands (e.g., RGD peptides, EGF) to enhance cell adhesion and proliferation. Overall, these future efforts will help translate the silk-cryoMN platform from proof-of-concept to the clinic.

## 5. Conclusions

The delivery of therapeutic cells to the gastric wall holds significant promise for treating gastric cancer, peptic ulcers, and other gastric disorders, yet remains challenging due to the lack of minimally invasive platforms that can both preserve cell viability during storage and penetrate the mechanically resilient gastric tissue. In this study, we developed a mechanically reinforced, porous silk fibroin-based cryogenic microneedle (silk-cryoMN) platform to address these challenges. Systematic optimization identified 1.5% (*w*/*v*) silk as the optimal concentration, yielding porous scaffolds with interconnected pores (mean diameter 24.4 ± 7.9 μm, porosity ~81%), high compressive strength (422.8 ± 73.4 MPa), and a 3.4-fold increase in β-sheet content relative to sol-silk. Micro-CT analysis further confirmed the isotropic, well-connected porous architecture with favorable permeability, supporting uniform cell loading (up to 3 × 10^6^ cells per scaffold) and efficient post-thaw release.

The silk-cryoMNs exhibited markedly superior thermal stability compared to H_2_O-cryoMNs, maintaining structural integrity for over 60 s at room temperature—a duration sufficient for practical handling. Cryopreservation medium optimization with 100 mM sucrose and 2% DMSO achieved post-thaw cell viabilities exceeding 80% for both human and murine cells after 11 days of cryopreservation, with the majority of viable cells released within 1 h. Notably, DMSO and sucrose were selected as permeable and impermeable vitrification agents, respectively, thereby enabling intracellular vitrification with DMSO and membrane stabilization with sucrose. Ex vivo proof-of-concept evaluation using porcine gastric tissue demonstrated that silk-cryoMNs achieve effective tissue penetration (penetration depth of 422–448 μm within 30 s) and successful release of revived cells, confirming the platform’s functional utility for gastric wall delivery.

Overall, our work provides silk-cryoMNs as a biocompatible, scalable, and mechanically robust platform for gastric-targeted cell therapy. By overcoming the critical translational barriers of gastric wall penetration, handling stability, and cell viability preservation, this work provides a minimally invasive alternative to conventional endoscopic needle injection and surgical transplantation. Further studies will center on in vivo evaluation in gastric disease models and the incorporation of bioactive motifs into the silk matrix to promote cell engraftment and therapeutic outcomes. However, we emphasize that this is an early-stage study; ex vivo results cannot be directly extrapolated to clinical use. Further studies will center on in vivo evaluation in gastric disease models, long-term safety assessment, and the incorporation of bioactive motifs into the silk matrix to promote cell engraftment and therapeutic outcomes.

## Data Availability

The original contributions presented in this study are included in the article/Supplementary Material. Further inquiries can be directed to the corresponding author.

## Supplementary Materials

The following supporting information can be downloaded at: https://www.mdpi.com/article/10.3390/polym18131654/s1, Figure S1: The stress–strain curves of the fully frozen porous silk scaffold at different concentrations. Figure S2: The confocal image of HeLa-eGFP cells; Figure S3: Maximum cell loading analysis; Figure S4: The confocal image of revived HeLa with with medium (0 mM) and different concentrations of sucrose; Figure S5: The confocal image of revived HeLa with different concentrations of DMSO; Figure S6: The SEM image of the revived human gastric mucosal epithelial cells (GES-1) and the revived mouse embryonic fibroblast (NIH-3T3) in silk-cryoMNs following cryopreservation for 11 days using an optimized cryopreservation medium (100 mM sucrose and 2% DMSO).
