# Supplementary material for: Mechanical-Enhanced Porous Silk-Based Cryogenic Microneedles for Cell Thawing/Revival in the Gastric Wall
Source: Polymers (Basel). 2026 Jul 3;18(13):1654. doi: 10.3390/polym18131654 (PMC13363972; doi:10.3390/polym18131654)
Supplement: Supplementary file 1 [file polymers-18-01654-s001.zip › polymers-4349965-supplementary.pdf]

Supplementary information

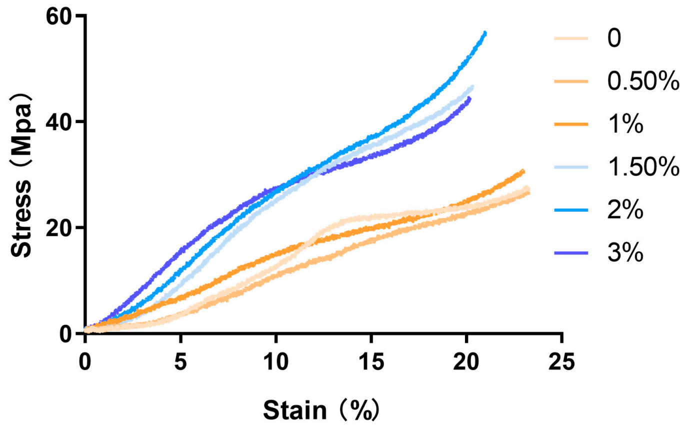

Figure S1. The stress–strain curves of the fully frozen porous silk scaffold at different concentrations. The data shown are from a representative sample.

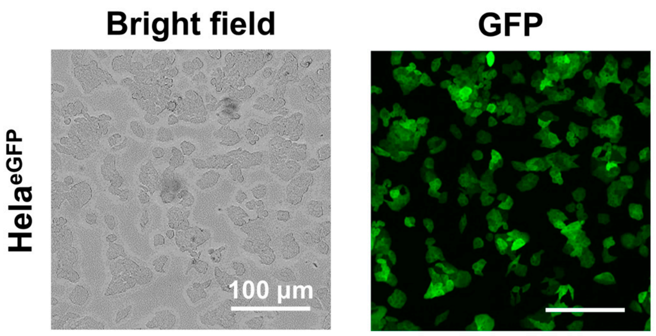

Figure S2. The confocal image of HeLa-eGFP cells, scale bar = 100 μm. The data shown are from a representative sample.

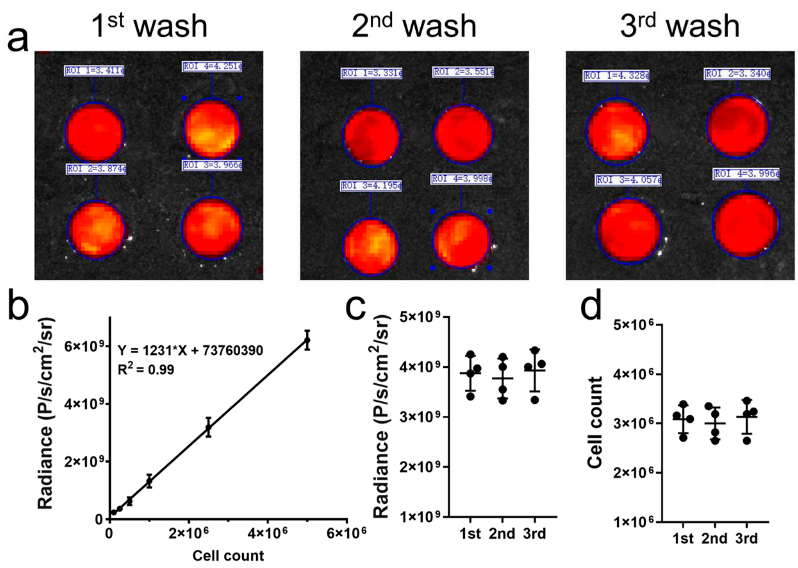

Figure S3. Maximum cell loading analysis. a. Macroscopic fluorescence imaging of silk porous scaffold loaded with HeLa-eGFP cells after first, second, and third wash in PBS. b. Quantification curve of cell count, n = 3. c, d. The fluorescence radiance and calculated cell count of scaffold loaded with HeLa-eGFP cells after 1st 2nd 3rd wash in PBS, n = 4. The data shown are from a representative sample and presented as means ± s.d.

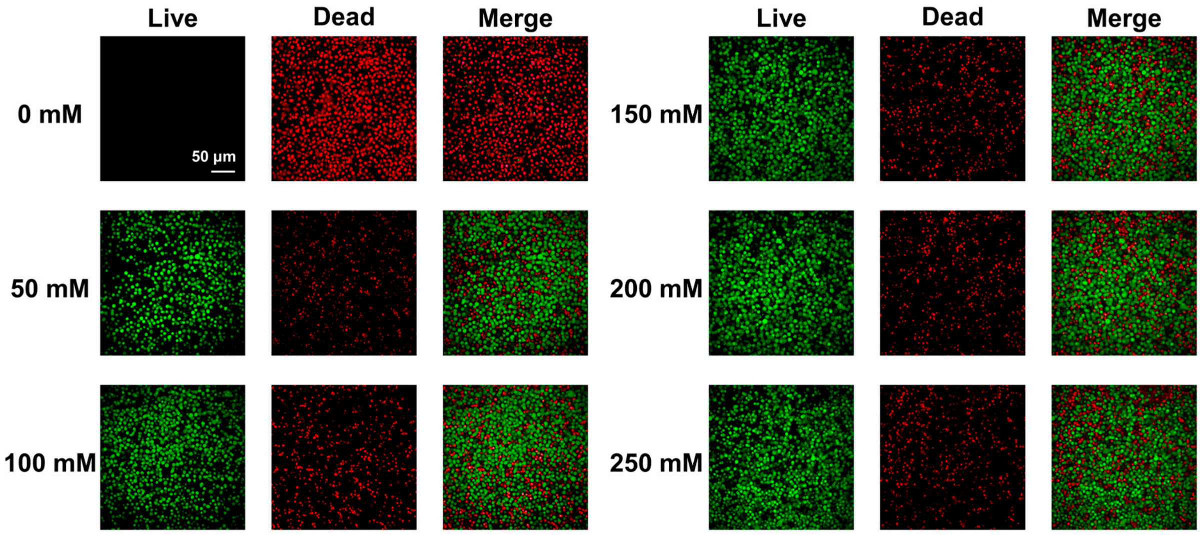

Figure S4. The confocal image of revived HeLa with medium (0 mM) and different concentrations of sucrose, scale bar = 50 μm. The data shown are from a representative sample.

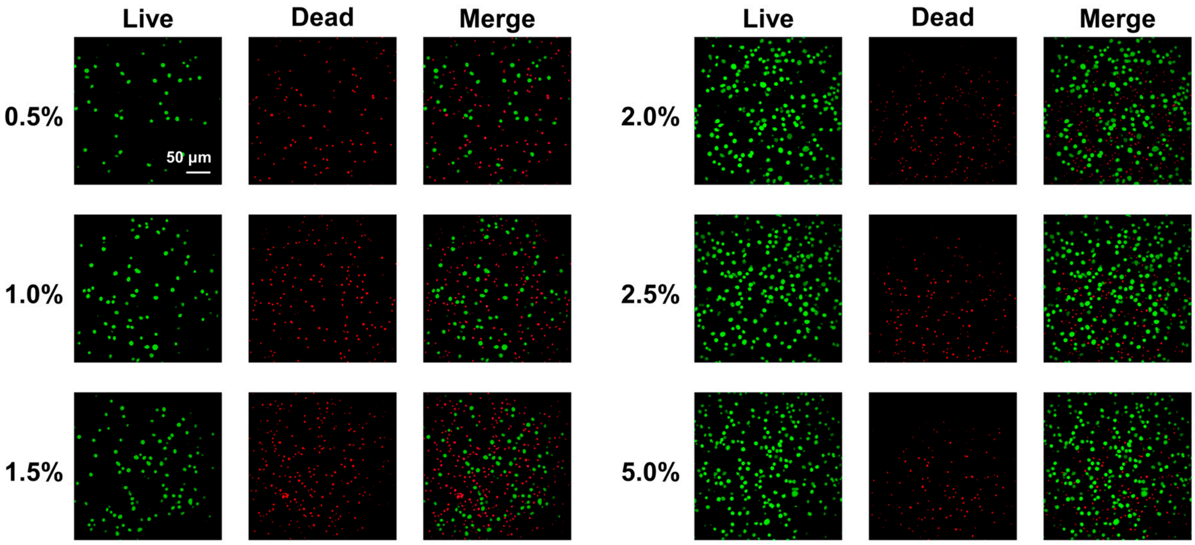

Figure S5. The confocal image of revived HeLa with different concentrations of DMSO, scale bar = 50 μm. The data shown are from a representative sample.

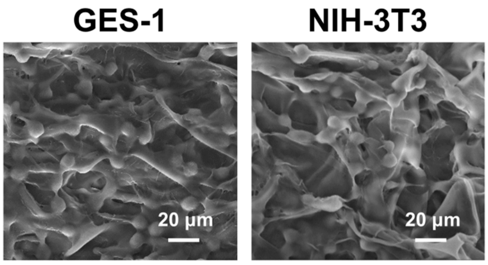

Figure S6. The SEM image of the revived human gastric mucosal epithelial cells (GES-1) and the revived mouse embryonic fibroblasts (NIH-3T3) in silk-cryoMNs following cryopreservation for 11 days using an optimized cryopreservation medium (100 mM sucrose and 2% DMSO), scale bar = 50 μm. The data shown are from a representative sample.
